# The rise of dietary diversity in coral reef fishes

**DOI:** 10.1098/rspb.2024.1004

**Published:** 2024-08-28

**Authors:** Isabelle Ng, David R. Bellwood, Jan M. Strugnell, Valeriano Parravicini, Alexandre C. Siqueira

**Affiliations:** ^1^ Research Hub for Coral Reef Ecosystem Functions, College of Science and Engineering, James Cook University, Townsville, Queensland 4811, Australia; ^2^ Centre for Sustainable Tropical Fisheries and Aquaculture, College of Science and Engineering, James Cook University, Townsville, Queensland 4811, Australia; ^3^ PSL Université Paris: EPHE-UPVD-CNRS, USR3278 CRIOBE, University of Perpignan, Perpignan 66860, France; ^4^ Institut Universitaire de France, Paris, France; ^5^ Centre for Marine Ecosystems Research, School of Science, Edith Cowan University, Joondalup, Western Australia 6027, Australia

**Keywords:** Bayesian phylogenetic comparative methods, evolutionary prey transitions, macroevolution, mass extinctions, multivariate ancestral state reconstruction, trophic ecology

## Abstract

Diet has been identified as a major driver of reef fish lineage diversification, producing one of the most speciose vertebrate assemblages today. Yet, there is minimal understanding of how, when and why diet itself has evolved. To address this, we used a comprehensive gut content dataset, alongside a recently developed phylogenetic comparative method to assess multivariate prey use across a diverse animal assemblage, coral reef fishes. Specifically, we investigated the diversification, transitions and phylogenetic conservatism of fish diets through evolutionary time. We found two major pulses of diet diversification: one at the end-Cretaceous and one during the Eocene, suggesting that the Cretaceous–Palaeogene mass extinction probably provided the initial ecological landscape for fish diets to diversify. The birth of modern families during the Eocene then provided the foundation for a second wave of dietary expansion. Together, our findings showcase the role of extinction rebound events in shaping the dietary diversity of fishes on present-day coral reefs.

## 1. Introduction

Animal diets represent an interplay among species’ behavioural, physiological and morphological traits [1,2] and are a major determinant of the flow of energy and nutrients in ecosystems. Beyond defining the ecological roles of species, diets also play a role in shaping broader macroevolutionary processes. For instance, in response to ecological opportunity, vertebrate assemblages have consistently demonstrated an expansion in ecomorphological space [1,3,4]. Evolutionary response to ecological opportunity has been shown to influence lineage diversification among various clades, with trophic evolution being identified as a major driver of lineage diversification in lizards [5], birds [6], mammals [7] and coral reef fishes [8,9]. However, despite a growing body of literature exploring how diets influence lineage diversification, the diversification of diet as a multivariate trait remains to be investigated over evolutionary time scales (but see [10]).

Fishes are the dominant consumers on coral reefs and represent an ideal focal group to examine patterns of dietary diversification. They are highly speciose and demonstrate a remarkable diversity of prey resources and feeding strategies [11–13]. Thus far, studies on the evolution of diets in coral reef fishes have mostly used discrete categorizations such as trophic groups (e.g. carnivore, herbivore and invertivore) or levels of specialization (e.g. specialist or generalist) [8,9,14]. These categorizations of diets have provided important insights into the role of trophic innovations as a major driver of reef fish lineage diversification [8,9,15]. However, the lack of a large-scale, quantitative compilation of specific gut content data has stymied the understanding of the drivers of diet evolution in this speciose vertebrate group. Indeed, the large number of potential trophic interactions on coral reefs requires high-resolution information capable of capturing the nuances associated with each dietary niche. Coral reef fish species often consume various prey items and prey proportions. Thus, by categorizing diet into univariate groups, we may not be capturing the true dietary niche breadth of a given species, resulting in the potential loss of fundamental ecological and evolutionary information. Fortunately, a global compilation of fish gut content data [13,16] provides a unique opportunity to address this issue and to explore the macroevolution of coral reef fish diets within a multivariate framework.

The main purpose of our study is to uncover the macroevolutionary patterns of diet diversification across coral reef fishes. We achieve this by combining the most comprehensive reef fish gut content dataset [13,16] to date, with well-sampled phylogenies of ray-finned fishes [17,18]. We also examine evolutionary diet states and prey transitions and assess whether these patterns are phylogenetically conserved. Our analysis revealed two major phases of diet diversification in coral reef fishes that were previously unknown, one at the Cretaceous–Palaeogene (K–Pg) boundary (66 million years ago (Ma)) and the other during the Eocene (56−34 Ma). These results provide the chronological context within which shifts in the diversification of coral reef fish lineages, morphologies and niches occurred across deep time.

## 2. Material and methods

### (a) Coral reef fish phylogenies

We used the phylogenetic tree containing 11 638 species of ray-finned fish (Actinopterygii) sourced from fishtreeoflife.org [18]. This phylogeny was constructed using a 27-gene multi-locus alignment and was subsequently time-calibrated using fossil data. Full details of the phylogenetic tree are given by Rabosky *et al.* [18]. We pruned the phylogeny using the *ape* R package [19] to maintain only species from the gut content dataset [13,16] and from the 13 consensus fish families characterized by their occurrence on coral reefs worldwide (sensu [20,21]). The families include the Acanthuridae, Apogonidae, Blenniidae, Carangidae, Chaetodontidae, Gobiidae, Holocentridae, Labridae, Lutjanidae, Mullidae, Pomacanthidae, Pomacentridae and Serranidae (sensu [20,21]).

The process described above was repeated with another phylogenetic tree to compare the results of our phylogenetic comparative analyses across two phylogenies with different dating schemes (electronic supplementary material, figures S1–S3). Following the approach of Siqueira *et al*. [22], the Actinopterygii tree [18] was recalibrated based on the backbone of a recent phylogenomic analysis of spiny-rayed fishes (Acanthomorpha) [17]. The Ghezelayagh *et al*. chronogram [17] was inferred using ultraconserved elements sampled from species of nearly all acanthomorph families. Even so, the species level sampling of the Acanthomorpha tree [17] was much lower relative to that of the Actinopterygii tree [18]. To ensure similar sampling levels between both trees, we applied the congruification approach [23], which involved selecting shared nodes at the genus level between both trees to create a reference tree. This reference tree was then used to recalibrate the Actinopterygii tree [18]. Specific information about this method is provided in Siqueira *et al*. [22]. This newly time-calibrated tree, however, contained divergence times that were generally younger and not in concordance with the fossil record. For this reason, we opted to use the original Actinopterygii phylogeny [18] herein and interpreted the results from the recalibration of the Acanthomorpha tree [17] as the earliest threshold of uncertainty around the timing of diet expansion (electronic supplementary material, figure S2).

### (b) Diet data

We compiled data on coral reef fish diets using the comprehensive gut content dataset from Parravicini *et al*. [13]. This dataset includes visual gut content data from adult individuals, collated from six locations: Marshall Islands [24], Puerto Rico and the Virgin Islands [25], Hawaii [26], Madagascar [27], Okinawa [28] and New Caledonia [13]. The total dataset included 13 961 fish guts containing ingested material from 615 fish species. To ensure the taxonomic nomenclature was up to date, verification of fish species and family names was conducted using the *rfishbase* R package [29] and subsequently cross-checked with the species from the phylogeny [13]. The original dataset contained 1200 individual prey items, categorized into 38 ecologically informative groups using higher level prey taxonomy, except for the following categories: benthic autotrophs (e.g. algae and seagrass), detritus, inorganic (e.g. sand) and zooplankton (i.e. gelatinous and non-gelatinous zooplankton and the eggs and larvae belonging to all taxa) [13]. All species included in this study had a sample of at least three individuals with non-empty guts.

The original gut content dataset, reported in both volumetric proportions and frequency of prey ingested material, was standardized into volumetric proportions [13]. This was calculated for each species by dividing the quantity of each prey group by the sum of all quantities. For species with data from more than one site, this process was calculated per site. This is because each site may have sampled different numbers of non-empty guts, which would influence the total counts calculated at the next stage. Volumetric proportions were then converted into prey item counts (i.e. full gut equivalents) to fit the Markov process model [30] designed for multivariate count data (see Phylogenetic comparative methods below). To do this, we multiplied the volumetric proportions by the total number of non-empty guts sampled from that given species and then rounded to the nearest integer (M. C. Grundler 21 March 2022, personal communication). Any zeroes in the dataset were subsequently removed. This conversion results in count data where every individual non-empty gut is full of one prey type, while still retaining the approximate volumetric proportions that were originally observed (M. C. Grundler 21 March 2022, personal communication).

Uncertainty due to uneven sampling across the gut content data has been explicitly accounted for by the model described in Grundler & Rabosky [30]. To test the efficacy of the model in accounting for sampling effort, we repeated the analysis using full stomach equivalent data based on the average number of non-empty guts across all samples. We did this by multiplying the volumetric proportions by the average number of non-empty guts across all species. The results were similar to those in the main text (electronic supplementary material, figures S4–S6), suggesting that the model does indeed account for sampling effort.

**Figure 1.**
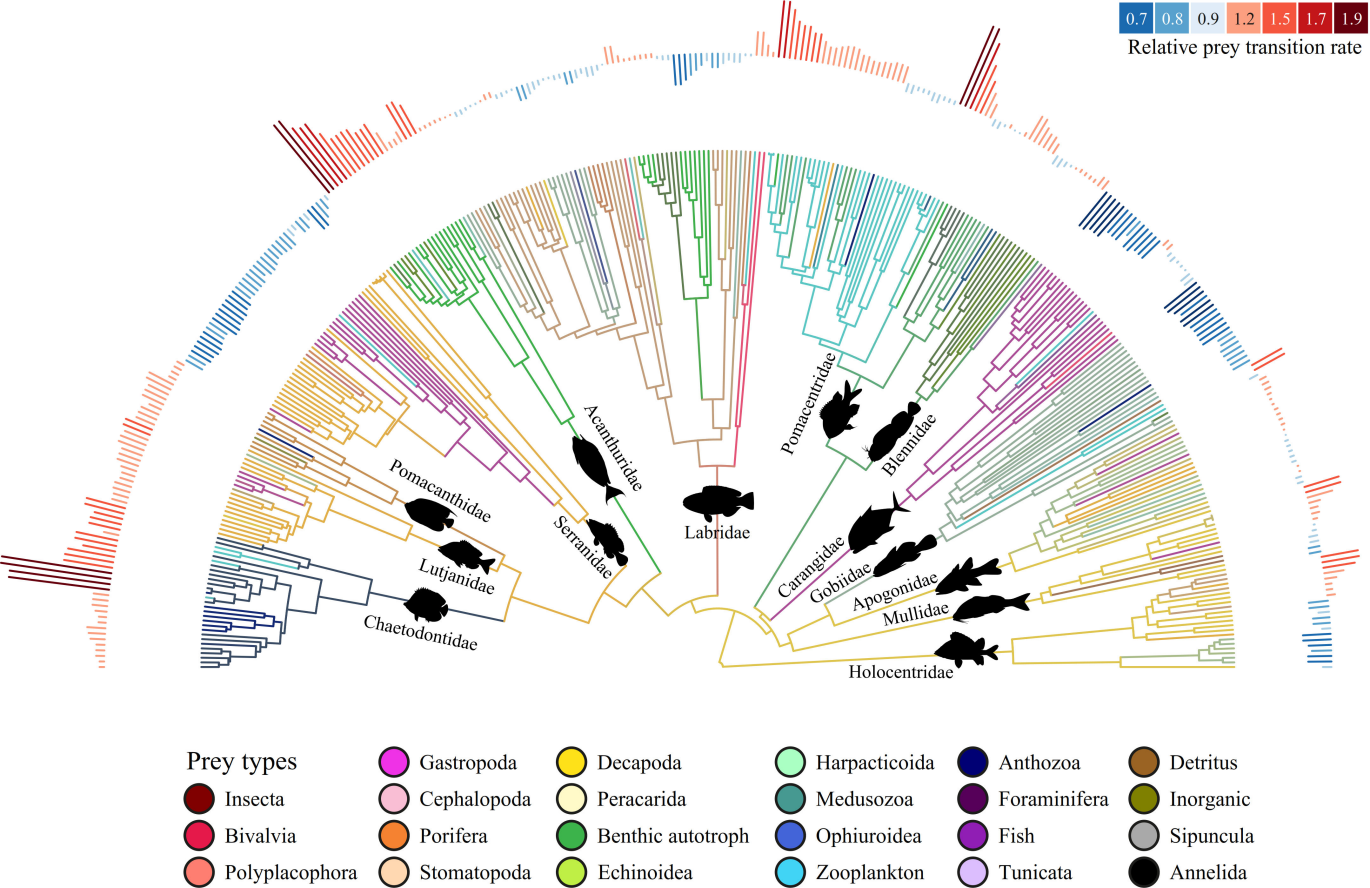
Ancestral reconstruction of diet states across the 13 coral reef fish families that occur on reefs globally. Each prey type was assigned a specific colour (bottom legend); therefore, branch colours represent the ancestral reconstructions of diet states through the model-inferred proportional mix of colours (i.e. prey). The red and blue bars above each tip represent the prey transition rates per species relative to the average prey transition rates of all coral reef fishes. The strength of the rates is represented by the relative length of the bar and the colour of the bar represents high (red) or low (blue) rates as seen in the top right legend. Fish silhouette graphics were sourced from Fishualize [31].

**Figure 2.**
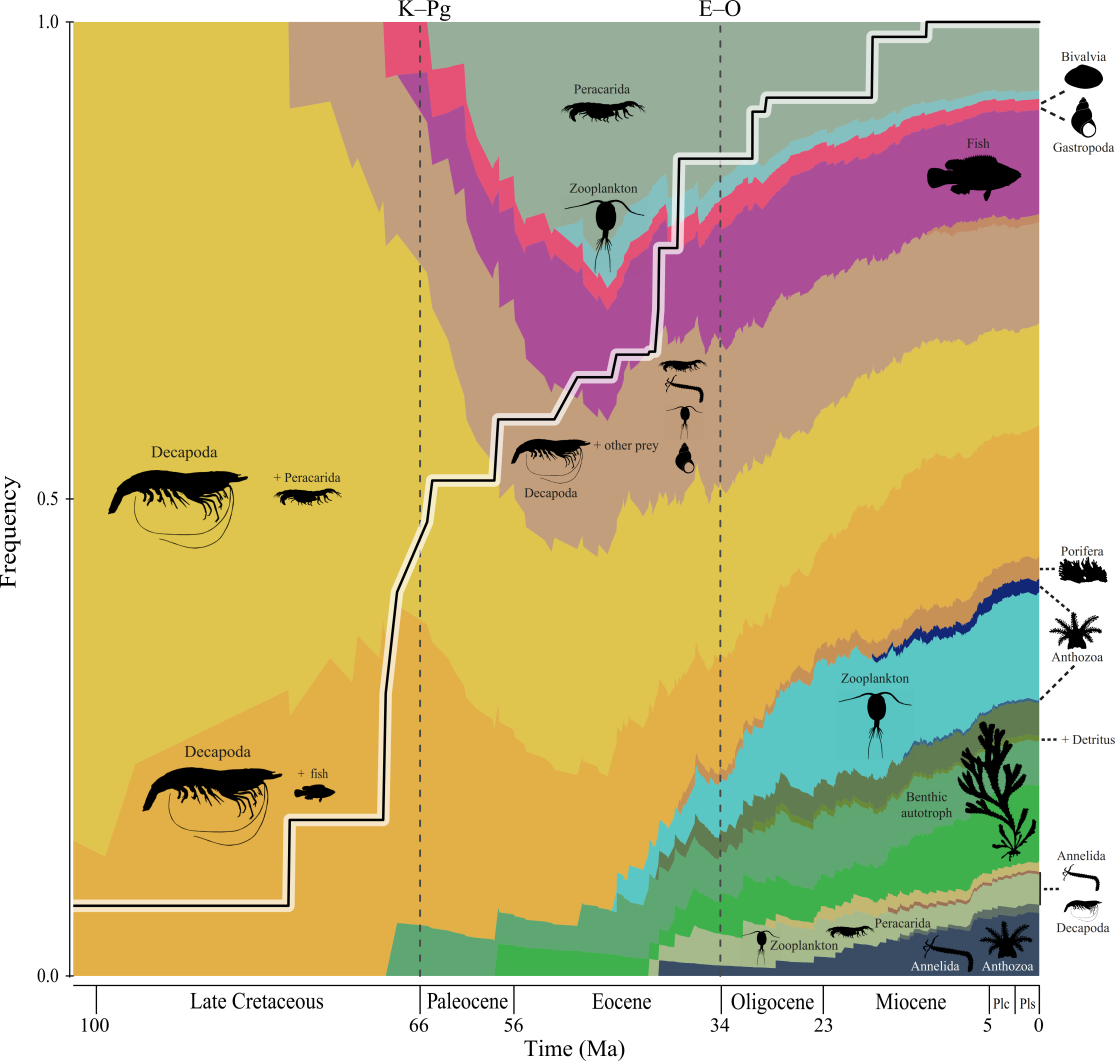
Proportional diet state expansion in coral reef fish lineages over evolutionary time. Each colour is a multivariate diet state made up of a proportional mix of prey types (colours in correspondence with figure 1) estimated by a multinomial probability distribution. The placement of the prey silhouettes represents the main prey type(s) (i.e. those with the highest multinomial probabilities) for the diet state that it is overlaying. For example, benthic autotroph (green) is the main prey type across the various green diet states in the bottom right corner. The solid black and white line represents the total among-lineage diet breadth, which was calculated as the sum of the greatest absolute difference in proportional use of a single prey type among the set of nodes at least the same age as the internal node over all prey categories. The dashed (vertical) lines represent the Cretaceous–Palaeogene (K–Pg) extinction event and Eocene–Oligocene (E–O) transition. Except for the Late Cretaceous, all time slices are grouped by geological epochs (Plc = Pliocene and Pls = Pleistocene) and units of time are expressed in million years ago (Ma). The origin of diet states is set by the crown clade age of the reconstructed ancestor (129.6 Ma) but was excluded from this figure to highlight the main time slices of dietary expansions (see electronic supplementary material, figure S21 for the full figure). The sample from the posterior with the highest probability for *K* = 1000 is displayed here with additional samples in electronic supplementary material, figure S19. Fish silhouettes are sourced from Fishualize [31] and all other silhouettes are public domain from PhyloPic (phylopic.org).

The merged phylogenetic and diet dataset included 349 species across the 13 focal families (figure 1) and 36 prey item categories. We reduced the number of infrequently consumed prey items by removing prey groups that demonstrated both a sum of ≤ 20 total counts (i.e. full gut equivalents) and ≤ 10 total number of observations across the entire dataset. This process narrowed down the number of prey categories to 23. This was done to improve the interpretability of the results (i.e. fewer colours to muddy the diet states; see Phylogenetic comparative methods below) and minimize potential biases in the diet categorizations. To verify our results based on 23 prey items, we ran the model with all 36 prey items and found similar results to those in the main text (electronic supplementary material, figures S7–S9). The only notable difference was the increase in the proportional representation of the ancestral diet over time (electronic supplementary material, figure S8) when compared with the results from 23 prey items (figure 2). However, this difference did not affect the main results of our study, which were to highlight the major expansions of dietary diversity across coral reef fishes. The timing and magnitude of these pulses in total among-lineage diet breadth remained roughly the same (figure 2; electronic supplementary material, figure S8), suggesting that removing the infrequently consumed prey items did not change our main findings.

The fish gut content dataset comes with a few limitations. Firstly, gut content data are based on ingested material and do not necessarily denote an individual’s true diet. This is evidenced in the existence of inorganic material as a prey category, which was probably a result of unidentified ingested material found in fish gut samples. Inorganic material nonetheless represents only a minor fraction of the total prey counts. Furthermore, all prey categories (including inorganic material) are items that can be categorized into defined reef fish trophic guilds [13]. Another limitation of the gut content dataset is that ingested material belonging to certain prey groups like Decapoda is easier to identify relative to other groups, such as benthic autotrophs (e.g. algae). Decapod prey may also be overrepresented in the gut content dataset because they require much more time to digest relative to other prey types [32]. These limitations may have resulted in varying levels of resolution across the prey categories within the dataset. This potential lack of resolution, however, is unlikely to have major effects on our results, given that we are describing events that have occurred across large temporal scales. Finally, there are disparities in the taxonomic sampling across the gut content dataset, most notably for the highly speciose cryptobenthic families (the Blenniidae and Gobiidae), which have been understudied due to their small sizes and cryptic nature [33]. If taxonomic sampling in the dataset was more representative of the species diversity in the Blenniidae and Gobiidae, we may expect a proportional increase in benthic autotrophs, detritus and crustacean (e.g. Decapoda, Peracarida) diet states [9,33]. Given that the focus of this study spans across evolutionary time scales, the lower taxonomic sampling of cryptobenthic fishes is unlikely to have had major effects on our main results regarding the timings of dietary expansions. However, this indeed highlights the need for future work to detail the gut contents of cryptobenthic reef fishes.

### (c) Phylogenetic comparative methods

#### (i) Ancestral state reconstruction

**Figure 3.**
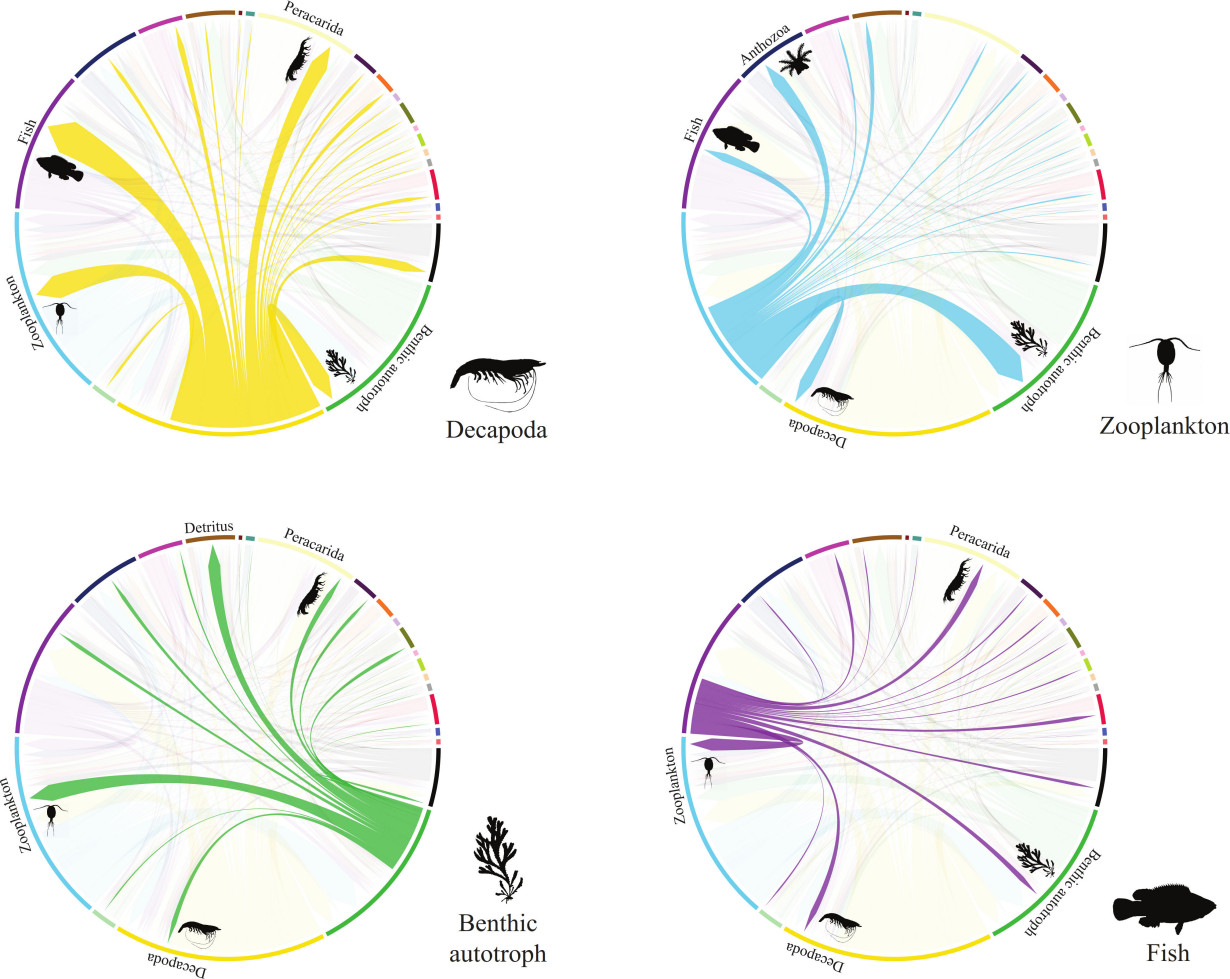
Evolutionary transitions from four of the prey groups with the highest average transitions to all other prey items. The thickness of the lines is proportional to the number of transitions and prey type colours correspond to those in the figure 1 legend. Prey silhouettes and labels along the edge of each circle represent the four most common prey items that each respective group has transitioned to. The fish silhouette was sourced from Fishualize [31] and all other silhouettes are public domain from PhyloPic (phylopic.org ).

We applied a recently developed Bayesian phylogenetic comparative method [30] that models the proportional use of discrete prey (or other resource) categories as a multinomial probability distribution. The Dirichlet-multinomial Markov model uses multivariate count data to estimate diet states by taking each gut content observation as a sample from the multinomial distribution. This means that every unobserved diet state is represented by a distinct multinomial distribution of proportional prey use. The Markov process allows for diet states to transition between the internal nodes of the tree, thus demonstrating how prey use has evolved over time. Colour was used as a tool to showcase the process of multivariate diet evolution [10]. To achieve this, each prey type is assigned a unique colour, and each diet state represents a proportional combination of prey types (i.e. colours) inferred from the model (figures 1–3). This method, implemented with the *macroevolution* R package, was initially developed by Grundler & Rabosky [30] and was subsequently applied to model diet evolution in snakes [10]. Here, we employed the same method using a phylogeny of extant species in addition to gut content data to reconstruct ancestral coral reef fish diets. Details on the full model theory and validation, and the visualization of diet evolution through colour are outlined in Grundler & Rabosky [10].

Following Grundler & Rabosky [10], we applied an uninformative Dirichlet prior (α = 1) to our model, along with a relatively high K value ( K = 1000). A high K value favours parsimony by applying stronger penalties to each transition in diet state. To test the effect of *K,* we ran the model with K = 22 (i.e. the mode number of diet states when K = 1000; see electronic supplementary material, figure S10) and with K = 500. For K = 22, the overall patterns remain unchanged (electronic supplementary material, figures S11–S13) compared with those with K = 1000 (figures 1–3). However, there was more uncertainty in the ancestral states (electronic supplementary material, figure S14), causing some posterior samples to favour an earlier appearance of zooplanktivory and a later occurrence of benthic autotroph-feeding (electronic supplementary material, figure S12). When using K = 500, the overall patterns also remained unchanged from the main results (electronic supplementary material, figures S15–S17), and there was minimal uncertainty in the ancestral states when using K = 500 (electronic supplementary material, figure S18) or K = 1000 (electronic supplementary material, figure S19). The model was run using a Gibbs sampler with 30 000 iterations along with a thinning interval of 10 and a warm-up period of 500 for a total of 3000 posterior samples. Likelihood and parameter traces validated model convergence (electronic supplementary material, figure S10).

We acknowledge that there may be some uncertainty around the ancestral diet state because coral reef fishes do not represent a monophyletic clade. Importantly, however, the main purpose of our study is not to describe the exact identity of the ancestral diet states at the root of the coral reef fish phylogeny, rather, our focus here is to examine the evolutionary patterns of dietary expansion. In essence, we are mainly interested in highlighting the evolution of the ecology of a group of fishes that share a common habitat.

#### (ii) Prey category transitions

An optimal transport model [10] was used to calculate the average number of evolutionary transitions between prey categories for each branch in the phylogeny. Transitions were computed through the amount of proportional prey item gains or losses required to transform an ancestral diet state into a descendant diet state. Estimated evolutionary transitions among prey categories were visualized with chord diagrams applied with the R package *circlize* [34]. Net transition rates were estimated per clade by taking the mean number of transitions among prey groups per clade and dividing it by the total branch length of all lineages per clade. The total number of transitions per clade was calculated by summing the total number of branch transitions and dividing it over all the branches descended from the clade’s root. The relative prey transition rates for the terminal node tips were inferred by taking the weighted mean of all clade rates from the top to the root of the phylogeny. Further information on the optimal transport model and how the evolutionary transitions were estimated can be accessed in Grundler & Rabosky [10].

#### (iii) Permutation test of diet expansion

We applied the null model of ecophenotypic diversification, which assumes that ecological opportunity is random across time and the phylogeny [10]. The results of this model were compared against the results from the Markov model to assess the statistical significance of the rate of dietary expansion (electronic supplementary material, figure S20). Specific details on this analysis can be found in Grundler & Rabosky [10]. All analyses were performed using the R statistical program [35].

## 3. Results

### (a) Ancestral reconstruction of diet states

The merged phylogenetic and diet dataset contained 349 species from the 13 fish families that occur on coral reefs globally (figure 1). The most common prey items found in the fish gut content dataset in both occurrence across species (*n* species) and total count across species (count) were—epibenthic Decapoda (*n* species = 183; count = 2747), zooplankton (e.g. eggs and larvae of all taxa) (158; 1350), benthic autotrophs (e.g. algae and seagrass) (154; 1394) and fish (145; 1320). The dominance of these items was also reflected in the ancestral reconstruction of coral reef fish diets (figure 1). The multivariate diet state of each lineage was typically dominated by the most common prey items per family: fish in the Carangidae and Serranidae; Anthozoa and Annelida in the Chaetodontidae; benthic autotrophs (e.g. algae) in the Acanthuridae, Blenniidae and Pomacentridae; Decapoda in the Apogonidae, Holocentridae, Labridae, Lutjanidae, Mullidae and Serranidae; Peracarida in the Apogonidae and Gobiidae; Porifera in the Pomacanthidae and zooplankton in the Gobiidae and Pomacentridae (figure 1). The reconstruction also suggested that the ancestral coral reef fish lineage had a dietary state dominated by Decapoda (but see Material and methods; figures 1 and 2).

Prey types are relatively conserved within families, with the exception of the Labridae (figure 1). Relative rates of prey switching (i.e. relative to the average rate across all coral reef fishes) are generally low in the Labridae, which suggests that prey items (e.g. Gastropoda, benthic autotrophs) tend to be conserved within larger clades in this family (figure 1). All lineages within the Chaetodontidae, Lutjanidae, Pomacanthidae and Acanthuridae, and most lineages in the Pomacentridae and Blenniidae demonstrated high relative rates of prey switching (figure 1). On the other hand, the Serranidae, Carangidae and Gobiidae exhibited low relative rates of prey switching (figure 1). A relatively even mixture of both high and low rates of diet evolution was observed in the remaining families: the Apogonidae, Mullidae and Holocentridae (figure 1). The highest recorded rates of prey switching were observed in the families Chaetodontidae, Acanthuridae and Pomacentridae, and the lowest recorded rates were found in the Carangidae and Gobiidae (figure 1).

### (b) Dietary expansion

By reconstructing the proportion of prey types in coral reef fish diets through time, we reveal that both the Cretaceous–Palaeogene (K–Pg, 66 Ma) and the Eocene (56−34 Ma) were closely linked with an expansion in the number of dietary states, as indicated by two pulses of total among-lineage diet breadth (figure 2). At, or around, the K–Pg boundary (66 Ma), there was a dramatic increase in the diversity of food items, involving fish and zooplankton (figure 2). From approximately 40 Ma to present-day, the rate of increase in the total among-lineage diet breadth across reef fishes was faster relative to the null model (see Material and methods; electronic supplementary material, figure S20). Across the Eocene, and especially in the late Eocene, there is a marked increase in dietary diversity, so that by the Eocene–Oligocene boundary (33.9 Ma), coral reef fish dietary states are relatively evenly dispersed (figure 2). At this stage, there is a greater prominence of benthic autotroph- (e.g. algae) and zooplankton-feeding, in addition to the appearance of new dominant dietary states such as Annelida, Anthozoa, Porifera, inorganic material and detritus (figure 2). In the early Miocene (23−16 Ma), a minor pulse in total among-lineage diet breadth coincides with the appearance and increased dominance of dietary states involving Annelida, Anthozoa and Porifera (figure 2). It is during this time that the total among-lineage diet breadth line is higher than expected (*p* < 0.05) when compared with the null model (see Material and methods; electronic supplementary material, figure S20). By the end of the Miocene (5.3 Ma), all multivariate diet states are established (figure 2). These results were consistent when we used an alternative phylogeny (electronic supplementary material, figures S1–S3) that was recalibrated based on a recent phylogenomic analysis of spiny-rayed fishes (Acanthomorpha; see Material and methods). In both analyses, the first large peak in among-lineage diet breadth took place during the K–Pg (figure 2; electronic supplementary material, figure S2). However, the analysis using the alternative phylogeny suggests that the second large peak in the total among-lineage diet breadth may have happened at a slightly younger age (electronic supplementary material, figure S2). This result was probably due to the younger ages from the recalibrated phylogeny (see Material and methods).

### (c) Evolutionary transitions among prey types

The number of transitions varies among prey types with some resource categories demonstrating greater turnover than others (figure 3). This suggests that the use of these prey groups probably differed across evolutionary time with regard to prey availability and predator adaptability. Of all prey types, Decapoda demonstrate the highest number of losses (i.e. transitions away from; figure 3), which is expected, given that it is the predicted ancestral state (but see Material and methods; figures 1 and 2; electronic supplementary material, S21). The highest average number of transitions is observed from Decapoda towards fish (figure 3). Decapoda consumers also transition frequently towards other prey including zooplankton, Peracarida, benthic autotrophs (e.g. algae) and Annelida (figure 3; electronic supplementary material, figure S22). Zooplankton-feeding lineages transition the most, on average, towards benthic autotroph-feeding, but are also observed to transition frequently to other groups such as epibenthic Decapoda, Anthozoa and fish (figure 3; electronic supplementary material, figure S22). Zooplankton demonstrates the highest number of gains, followed by benthic autotrophs and fish, suggesting that these are the most common prey transition destinations (electronic supplementary material, figure S22). Overall, coral reef fishes demonstrate the highest transitions away from decapod-feeding, and the highest transitions towards zooplanktivory.

## 4. Discussion

The trophic diversity of modern coral reef fishes is the result of two major evolutionary events of dietary expansion—one close to the Cretaceous–Palaeogene (K–Pg) boundary and another during the Eocene (figure 2). Although the precise timing of these observed pulses in dietary diversification may be hard to infer from phylogenetic data alone (electronic supplementary material, figure S19), they nevertheless largely coincide with periods of elevated biological turnover [36–42], regardless of the phylogeny used to conduct the analysis (electronic supplementary material, figures S1–S3). Furthermore, given the magnitude of change in dietary diversity at the end-Cretaceous, a direct link with the K–Pg seems most likely (figure 2; electronic supplementary material, figure S2). The first major bout of dietary diversification appears to have occurred concomitantly to a shift in marine habitat structure that accompanied the K–Pg transition. This suggests that rebounds after extinction events were important in shaping the diet diversity found on modern coral reefs.

While the K–Pg boundary is most notable for the extinction of non-avian dinosaurs, the marine realm also suffered drastic losses of evolutionary lineages [43]. The extinction of previously dominant assemblages such as ammonites, and the earlier loss of rudists [44], transformed marine landscapes from low diversity rudist reefs of the Cretaceous to modern algal- and scleractinian-dominated systems of the early Cenozoic [45]. Thus, it seems reasonable to infer that the K–Pg rebound event triggered dietary diversification in coral reef fishes (figure 2), and this was probably underpinned by two main factors. Firstly, the extinction of Mesozoic fish lineages and ammonites meant that these old trophic niches became vacant for surviving lineages to fill. For example, the K–Pg event led to a marked decline in plankton diversity and a dramatic loss of pelagic piscivorous fishes; these events were probably interconnected [46]. Secondly, the origination of new taxa [17,47] and the formation of new dominant benthic groups [45] resulted in the availability of new niches. The post K–Pg dietary expansion in coral reef fishes may also have been facilitated by the appearance of new prey species that were, likewise, expanding into available niche space. For example, bivalve and gastropod molluscs radiated shortly after the K–Pg [39,48–51]. Hence, shifts in predator–prey relationships and the diversification of fish diets could have been a response to the abundance and diversity of their prey.

The dietary diversification of coral reef fishes (figure 2) is also mirrored by the expansion of teleost fish feeding modes after the K–Pg, such as the rise in benthic feeding [52,53] and an ongoing expansion in jaw protrusion [54]. Indeed, morphological diversification across a wide range of marine teleost fishes was also noted after the K–Pg [17,55,56]. Our results strongly suggest that the initial dietary expansion in coral reef fishes was closely linked with both the morphological diversification of teleost fishes and ecological release following the K–Pg mass extinction event.

In the Palaeocene–Eocene (66−33 Ma), following the K–Pg event, we see the expansion of Acanthomorpha [17,47] and Actinopterygii [41] fish lineages. This includes the birth of modern reef fish families [20,57,58], the emergence of ecological functions [59] related to the rise of herbivory [15], nocturnal feeding and high-precision benthic feeding [60]. This geologically warm time was also characterized by the evolution of faster growth rates and smaller body sizes in many reef fish lineages [22], which probably enabled fishes to exploit a greater diversity of habitats and prey resources. The rise of piscine herbivory [52,59,61] in particular, could have also contributed to the Eocene dietary expansion, with the proportion and diversity of benthic autotroph diets gradually increasing throughout this Epoch (figure 2). This combination of new lineages with novel functional capabilities may have underpinned the second major expansion of dietary niche diversity in the Eocene, with the strongest increase being close to the Eocene–Oligocene transition (figure 2). Though quantitatively not as significant as the K–Pg mass extinction, the Eocene–Oligocene transition was the result of a widespread cooling of the Earth [62]. The Eocene–Oligocene was also associated with the geological events that led to the ‘hopping’ of the marine biodiversity hotspot from the Tethys Sea to the Indo-Australian Archipelago [63] and the initiation of modern reef structures [64]. At this time, reef fish lineages displayed signs of a cryptic extinction event, although rapid cladogenesis took place thereafter [37]. There may have been an interplay between these events and the steep ascent of the total among-lineage diet breadth observed at the end-Eocene (figure 2).

Our findings suggest a temporal misalignment between diet, lineage and functional expansions in coral reef fishes. Dietary diversification occurred in the Palaeocene and Eocene (figure 2), prior to trophic specializations, and functional and lineage divergence in the Miocene [59,65]. Indeed, we only observed four new diet states from the Miocene to present-day (23−0 Ma) (figure 2). Furthermore, a predominantly benthic autotroph (e.g. algae) diet state first appeared close to the K–Pg and continued to diversify, mostly during the Eocene and up until the Oligocene (34−23 Ma; figure 2). This largely coincides with the fossil record that traces the origins of piscine herbivory to the early Eocene [66]. Trophic expansion in the Eocene (figure 2) probably granted coral reef fishes the dietary basis in which they could functionally innovate from. The effect of this was seen in the proportional expansion of a benthic autotroph-dominated diet from the Eocene all the way to the Pleistocene (figure 2).

Although the Eocene was important for the diversification of coral reef fish diets, it is during the Miocene that the evolution of modern coral reefs takes place (phase 5 [59]). The Miocene is characterized as a period of lineage accumulation in reef-associated fishes [65] and of high functional and trophic innovation [8,15], possibly related to the colonization of high turnover reef flats [66]. For example, key ecosystem functions (e.g. bioerosion and turf, sediment and macroalgae removal) among herbivorous reef fish lineages diversified predominantly between the Miocene and Pleistocene [67]. Furthermore, a shift in morphospace towards features such as long teeth, high fin aspect ratios and increased distance between the eyes and mouth of herbivorous fishes enabled specialization within pre-existing trophic categories (e.g. specialized detritivory and algal-turf feeding) during the Miocene [8,66,68]. From this perspective, the Eocene was a period where dietary categories were fully established and the Miocene was a period of functional specialization [8,66], with this later bout of trophic innovations underpinning the highest lineage diversification rates in coral reef fishes [9]. In essence, dietary diversity was probably a necessary first step prior to the trophic specialization and, functional and lineage diversification in coral reef fishes [cf. 69].

With the origins of many modern coral genera [59] and the expansion of scleractinian-dominated reefs in the Miocene [45], coral-associated fish groups such as *Chromis* (F. Pomacentridae), *Apogon* (F. Apogonidae) and *Chaetodon* (F. Chaetodontidae), also started to diversify [37,65,70]. Our results support these earlier findings, with a marked proportional increase in Anthozoan diets (i.e. sea anemones and corals) during the Miocene (figure 2), mirroring the rise and expansion of corallivory in the Chaetodontidae [61,70].

While we found regular acquisitions of new feeding modes through evolutionary time, many coral reef fishes also transition between specific prey groups, especially to and from zooplanktivory (figure 3). The importance of zooplankton in coral reef fish evolutionary transitions has been previously observed when using broader trophic guilds [9]. The same patterns were found in individual reef fish families [71], such as the Pomacentridae [14] and the Lutjanidae [72]. This highlights that both fine- and coarse-scale analyses identified transitions to zooplanktivory as the most common among coral reef fishes. The prevalence of zooplanktivory as an evolutionary transition destination in reef fishes may be related to their ubiquitous reliance on plankton for nutrition during larval and juvenile stages [73].

Despite our fine-scale categorization of diets, we observe extensive phylogenetic niche conservatism across coral reef fish families (figure 1), i.e. the retention of similar multivariate diet states among lineages of the same family [74]. The idea of phylogenetic niche conservatism of diets has been contested, with evidence of diet lability in avian assemblages [75] and limited evidence of diet conservatism in primates [76] and mammals more broadly [77]. In contrast, strong evidence of phylogenetic conservatism has been observed in the diets of snakes [10], hexapods [78], tetrapods [79], and even more widely, across the animal tree of life [80]. Phylogenetic niche conservatism in tetrapods was observed along deep time scales (approx. 350 Ma) and across multiple variables— including diet—such as climate, body temperature, diel activity and habitat [79]. It is therefore unsurprising that a phylogenetic signal exists across the diets of most coral reef fish families (figure 1). The only exception is in the Labridae, which exhibits diet lability (figure 1), reflecting the high variability and specialization in their morphologies and feeding modes [15,81–84]. This degree of phylogenetic conservatism in coral reef fish diets can inform ecological patterns such as the structure of food webs [85] and highlight the disconnect between divergence and abrupt diversification, which has been shown to be underpinned by colour or reproduction [86].

In conclusion, our findings showcase temporal discontinuities in the macroevolution of coral reef fish diets, supporting the notion that diet diversification precedes trophic specialization and functional and lineage diversification [69]. We highlight the potential role of mass extinction rebound events in shaping present-day coral reef fish ecology and note the prevalence of phylogenetic conservatism across deep time scales. These findings underscore the value of investigating diet as a multivariate state across macroevolutionary time scales, and the role of evolutionary history in shaping extant high diversity ecosystems.

## Data Availability

The dataset used was synthesized by Parravicini *et al.* [13] and can be accessed on GitHub (github.com/valerianoparravicini/Trophic_Fish_2020) or in the Zenodo Repository for this study [87]. The original code for the models and figures was written by Grundler & Rabosky [10,30] and can be accessed in the Dryad Repository [88] and in the Zenodo Repository [89]. The data and modified script files used for this study can be found in the Zenodo Repository [87]. Supplementary material is available online [90].
